# Mixed diversity of shifting IOD and El Niño dominates the location of Maritime Continent autumn drought

**DOI:** 10.1093/nsr/nwaa020

**Published:** 2020-02-13

**Authors:** Chundi Hu, Tao Lian, Ho-Nam Cheung, Shaobo Qiao, Zhenning Li, Kaiqiang Deng, Song Yang, Dake Chen

**Affiliations:** School of Atmospheric Sciences, and Guangdong Province Key Laboratory for Climate Change and Natural Disaster Studies, Sun Yat-sen University, China; State Key Laboratory of Satellite Ocean Environment Dynamics, Second Institute of Oceanography, Ministry of Natural Resources, China; Southern Marine Science and Engineering Guangdong Laboratory (Zhuhai), and Key Laboratory of Tropical Atmosphere-Ocean System (Sun Yat-sen University), Ministry of Education, China; State Key Laboratory of Satellite Ocean Environment Dynamics, Second Institute of Oceanography, Ministry of Natural Resources, China; School of Atmospheric Sciences, and Guangdong Province Key Laboratory for Climate Change and Natural Disaster Studies, Sun Yat-sen University, China; Southern Marine Science and Engineering Guangdong Laboratory (Zhuhai), and Key Laboratory of Tropical Atmosphere-Ocean System (Sun Yat-sen University), Ministry of Education, China; School of Atmospheric Sciences, and Guangdong Province Key Laboratory for Climate Change and Natural Disaster Studies, Sun Yat-sen University, China; Southern Marine Science and Engineering Guangdong Laboratory (Zhuhai), and Key Laboratory of Tropical Atmosphere-Ocean System (Sun Yat-sen University), Ministry of Education, China; Institute of Environment, Energy and Sustainability, The Chinese University of Hong Kong, China; Southern Marine Science and Engineering Guangdong Laboratory (Zhuhai), and Key Laboratory of Tropical Atmosphere-Ocean System (Sun Yat-sen University), Ministry of Education, China; Department of Earth Science, University of Gothenburg, Sweden; School of Atmospheric Sciences, and Guangdong Province Key Laboratory for Climate Change and Natural Disaster Studies, Sun Yat-sen University, China; School of Atmospheric Sciences, and Guangdong Province Key Laboratory for Climate Change and Natural Disaster Studies, Sun Yat-sen University, China; Southern Marine Science and Engineering Guangdong Laboratory (Zhuhai), and Key Laboratory of Tropical Atmosphere-Ocean System (Sun Yat-sen University), Ministry of Education, China; Institute of Earth Climate and Environment System, Sun Yat-sen University, China; Southern Marine Science and Engineering Guangdong Laboratory (Zhuhai), and Key Laboratory of Tropical Atmosphere-Ocean System (Sun Yat-sen University), Ministry of Education, China; State Key Laboratory of Satellite Ocean Environment Dynamics, Second Institute of Oceanography, Ministry of Natural Resources, China

## Abstract

The Maritime Continent is a huge heat source region over the Indo-Pacific warm pool and it plays a key role in global weather/climate variations. The locations of Maritime Continent autumn droughts, linked to frequent rampant forest wildfires, are closely related to the mixed diversity of El Niño and Indian Ocean Dipole events.

Climate-change impact evaluation on Maritime Continent (MC) land precipitation is becoming an important research arena, given the severe forest fires induced by frequent autumn droughts under greenhouse warming [1,2] and the importance of latent heat released from MC-rainfall processes for local and global atmospheric circulations [1]. It is widely accepted that MC-precipitation activities, via changing Walker circulation [3], are indirectly modulated by El Niño/Southern Oscillation (ENSO) [1,4] and Indian Ocean Dipole (IOD) [5], which are both high-impact ocean–atmosphere coupled phenomena with a global ‘footprint’ on interannual timescales. Thus, their interaction attracts considerable scientific attention [3].

However, understanding of the changing impacts from shifting ENSO and IOD diversities on the MC land precipitation remains insufficient. Projection of the effects of ENSO–IOD change on the MC-drought positions is therefore inherently uncertain and is a subject of profound scientific interest in the present study. Here, the results show that, for the satellite era of 1979–2016, two distinct MC-drought positions are significantly modulated by different ENSO–IOD flavors during boreal autumn (i.e. the seasonal mean of September–November, SON).

Considering that the MC land precipitation possesses large dry–wet annual cycle and significant local features/variances due to the unique geographic location [1], here we apply a rotated empirical orthogonal function (REOF; see Supplementary Data and Methods for more details) to capture the interannual leading modes of the normalized-and-detrended NOAA (i.e. the National Oceanic and Atmospheric Administration) land-precipitation anomalies in the MC region (95°E–145°E, 11°S–9°N), which (i.e. the REOF1 and REOF2 shown in Fig. 1a and b, and see Supplementary Fig. 1 for details) highlight the rainfall deficit over the western MC (WMC) and the eastern MC (EMC), respectively. The corresponding principal components (i.e. RPC1 and RPC2) of MC land precipitation, with clear interannual variations, are plotted in Fig. 1a and b (*black lines*), which explain 34.3% and 27.9% of the total normalized variances (see Supplementary Note 1 and Supplementary Fig. 1 for details). It is worth noting that similar results can be reproduced with another three different sets of higher-resolution data (Supplementary Figs 2–4), suggesting that such REOF results are significant and independent of data choice.

**Figure 1.**
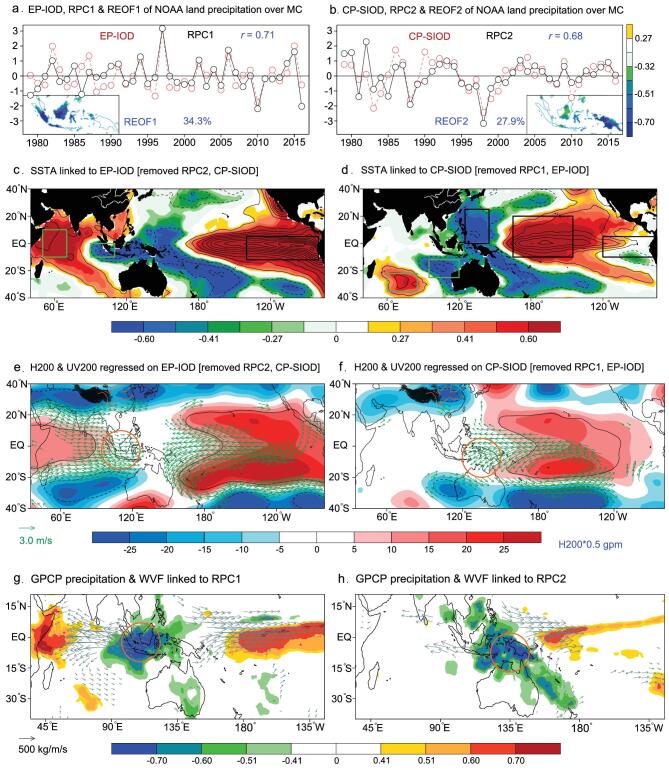
Modulations of different ENSO-IOD combinations on the MC precipitations in SON. Shown in (a) and (b) is the normalized time series (*black*) of RPC1 and RPC2 of NOAA land-precipitation anomalies over the MC (95°E–145°E, 11°S–9°N) and their REOF correlation modes during boreal autumn (SON) for 1979–2016 (see Supplementary Fig. 1 for details of REOF-correlation modes). Also shown in (a) and (b) are the normalized EP-IOD and CP-SIOD indices (*red* lines), respectively. Correlation (RPC1, EP-IOD) = 0.71 and correlation (RPC2, CP-SIOD) = 0.68. Shown in (c) and (d) are the SST-anomaly modes (*including correlation in shadings and regression in contours with an interval of 0.1 K*) associated with (c) EP-IOD index (*after removing RPC2 & CP-SIOD*) and (d) CP-SIOD index (*after removing RPC1 & EP-IOD*), respectively. Shown in (e) and (f) are the regressions of H200 (*shadings*, *P < 0.01 outlined by black lines*) and UV200 (*only the vectors withP < 0.01*) anomalies on the EP-IOD (*after removing RPC2 & CP-SIOD*) and CP-SIOD (*after removing RPC1 & EP-IOD*), respectively. Note that here the RPC2 & CP-SIOD or RPC1 & EP-IOD have been linearly removed from all variables before producing (c)–(f) due to the weak but significant correlations among them (see Supplementary Fig. 6). To obtain the large-scale land–ocean precipitation anomaly modes and to further cross-validate the reliability of the REOF results shown in (a) and (b), the anomaly patterns of GPCP v2.3 precipitation (correlation in *shadings, red lines indicate the robust |correlations| > 0.60*) and water-vapor flux (WVF, regressions, *only show the vectors with P < 0.01*) linked to RPC1 and RPC2 are also shown in (g) and (h), respectively. The green and black boxes in (c) and (d) outline the regions that define the EP-IOD and CP-SIOD indices (*see the text for details*), respectively. The orange circles in (e)–(h) highlight the relative MC position as a reference system.

The changes of Indo-Pacific sea-surface temperature (SST) related to RPC1 and RPC2 are characterized by two types of El Niño in the Pacific and two flavors of IOD in the Indian Ocean (Supplementary Fig. 5), respectively. Specifically, WMC droughts show strong linkages to the traditional combination of eastern Pacific (EP) El Niño and tropical IOD (hereafter EP-IOD; Supplementary Fig. 5a), whereas EMC droughts are mainly jointly induced by a new set of central Pacific (CP) El Niño and subtropical IOD (SIOD, hereafter CP-SIOD; Supplementary Fig. 5b). Then, the EP-IOD and CP-SIOD indices are accordingly defined in the following *Definition Description* and are shown in Fig. 1a and b (*red lines*), respectively.

***Definition description:*** The CP El Niño index (CPI) is calculated by [SST]_C_ – 0.5^*^[SST]_E_ – 0.5^*^[SST]_W_, where [SST]_C_, [SST]_E_ and [SST]_W_ represent the seasonal area-mean of monthly grid-normalized SST anomalies over the three regions: (165°E–145°W, 10°S–20°N), (120°W–70°W, 10°S–5°N), (125°E–145°E, 0°–25°N), respectively. The EP El Niño index (EPI) is defined as the seasonal area-mean of monthly grid-normalized SST anomalies over (140°W–80°W, 12°S–5°N). Likewise, the IOD index [6] is defined as [SST]_(50°E__–__70°E, 10°S–10°N)_ – [SST]_(90°E–110°E, 10°S–0°),_ whereas the SIOD index is defined as [SST]_(55°E–70°E, 25°S–15°N)_ – [SST] _(95°E–__120°E, 25°S–12°S)_. Finally, the EP-IOD and CP-SIOD indices are straightforwardly calculated from the normalized (EPI+IOD)/2 and (CPI+SIOD)/2, respectively (Supplementary Note 2). Relevant SST regions are outlined in Fig. 1c and d. Their correlations are shown in Supplementary Fig. 6.

As expected, their time series are strongly in phase, with high correlations (Fig. 1a and b) up to 0.71 (0.68) between RPC1 and EP-IOD (between RPC2 and CP-SIOD), statistically exceeding the 99.9% confidence level. Relevant SST modes are shown in Fig. 1c and d, which perfectly mirror the SST patterns (Supplementary Fig. 5) associated with RPC1 and RPC2, respectively. Two such types of combined Indo-Pacific SST modes would lead to different changes in (i) the large-scale Rossby waves (Fig. 1e and f) via upper-level divergence perturbed by tropical convective activities [7,8] and (ii) the low-level winds and convergence via forcing different surface-pressure gradients [9] (Supplementary Fig. 7a and b), which together result in distinct Walker-circulation anomalies with different ascending and sinking motions over the Indo-Pacific Ocean and the WMC/EMC (Supplementary Fig. 8), respectively.

In comparison with Fig. 1e and Supplementary Fig. 7a for EP-IOD events, Fig. 1f and Supplementary Fig. 7b reveal that the resultant upper-level convergent winds and low-level divergent winds are shifted from WMC to EMC during CP-SIOD events (referring to the orange circles shown in Fig. 1e–h), corresponding to the location changes in the sinking motions of the Walker circulation (see the red boxes shown in Supplementary Fig. 8a and b). Then, the resultant water-vapor-flux responses (see the orange circles shown in Fig. 1g and h and Supplementary Fig. 7c and d) contribute to the location shift of MC drought (Fig. 1g and h and Supplementary Fig. 7c and d). Such phenomena indicate the geographical adaptability of MC precipitation/drought to ENSO–IOD diversity (Supplementary Fig. 5). Of note is that Fig. 1c–f and Supplementary Figs 7 and 8 are obtained independently from the REOF analysis, except for the SST-box choices used for defining the EP-IOD and CP-SIOD indices, suggesting that the above results are reliable and robust.

In summary, the nature of ENSO–IOD combinations and the changes in their performances, including the modulation of tropical waves, convections and Walker-cell patterns, make good sense to understand MC-rainfall and forest-fire activities. Additionally, a higher prevalence of extreme El Niño and IOD events is anticipated in future climate scenarios [10,11] and that the spurious IOD as well as the Modoki El Niño are mysteriously changing under greenhouse warming [3,8,12], indicating that climate change and variability may exert more severe impacts on the MC autumn drought than previously thought. More importantly, our results highlight that, once the precursor signals of variant ENSO–IOD combinations were monitored or predicted, it would provide early warning to relevant policymakers to plan and act effectively to minimize forest-fire losses (including homes and crops destroyed, fisheries ruined, etc.) and shelter air quality and life safety during the dry-season months for MC countries.

## Supplementary Material

nwaa020_Supplemental_Files

## References

[1] Zhang T , Yang S, Jiang X et al. J Clim 2016; 29: 8871–9.

[2] Shawki D , Field RD, Tippett MK et al. Geophys Res Lett 2017; 44: 9996–10005. 32803204 10.1002/2017GL073660 PMC7427816

[3] Zhang W , Wang Y, Jin F-F et al. Geophys Res Lett 2015; 42: 8570–6.

[4] Jia X , Ge J, Wang S. Int J Climatol 2016; 36: 3384–97.

[5] Zhang L , Du Y, Cai W. Sci Bull 2018; 63: 1170–2. 10.1016/j.scib.2018.08.001 36751083

[6] Saji NH , Goswami BN, Vinayachandran PN et al. Nature 1999; 401: 360–3. 16862108 10.1038/43854

[7] Sardeshmukh PD , Hoskins BJ. J Atmos Sci 1988; 45: 1228–51.

[8] Hu C , Yang S, Wu Q et al. Nat Commun 2016; 7: 11721. 27251873 10.1038/ncomms11721 PMC4895717

[9] Lindzen RS , Nigam S. J Atmos Sci 1987: 44: 2418–36.

[10] Cai W , Santoso A, Wang G et al. Nature 2014; 510: 254–8. 24919920 10.1038/nature13327

[11] Wang G , Cai W, Gan B et al. Nat Clim Change 2017; 7: 568–72.

[12] Yang S , Li Z, Yu J-Y et al. Natl Sci Rev 2018; 5: 840–57.

