# Supplementary material for: Mixed diversity of shifting IOD and El Niño dominates the location of Maritime Continent autumn drought
Source: Natl Sci Rev. 2020 Feb 13;7(7):1150–3. doi: 10.1093/nsr/nwaa020 (PMC8289024; doi:10.1093/nsr/nwaa020)
Supplement: nwaa020_Supplemental_Files [file nwaa020_supplemental_files.docx]

Supplementary Data

**Mixed diversity of shifting IOD and El Niño dominates the location of Maritime Continent autumn drought**

Chundi Hu^1,2,3^, Tao Lian^2^, Ho-Nam Cheung^1,3^, Shaobo Qiao^1,3^, Zhenning Li^4,3^, Kaiqiang Deng^5,1^,

Song Yang^1,3,6,^* and Dake Chen^3,2,^*

^1^ School of Atmospheric Sciences, and Guangdong Province Key Laboratory for Climate Change and Natural Disaster Studies, Sun Yat-sen University, China

^2^ State Key Laboratory of Satellite Ocean Environment Dynamics, Second Institute of Oceanography, Ministry of Natural Resources, China

^3^ Southern Marine Science and Engineering Guangdong Laboratory (Zhuhai), and Key Laboratory of Tropical Atmosphere-Ocean System (Sun Yat-sen University), Ministry of Education, China

^4^ Institute of Environment, Energy and Sustainability, The Chinese University of Hong Kong, China

^5^ Department of Earth Science, University of Gothenburg, Sweden

^6^ Institute of Earth Climate and Environment System, Sun Yat-sen University, China

**SUPPLYMENTARY DATA**

- **Supplementary Data and Methods**
- **Supplementary Notes 1–2**
- **Supplementary References**
- **Supplementary Tables S1–3**
- **Supplementary Figures S1–9**

**Supplementary Data and Methods**

All monthly precipitations, atmospheric and oceanic datasets used in this study are listed in **Supplementary Table S1**. Note that the monthly mean SST data used in this study is averaged from the two datasets: (1) the NOAA Extended Reconstructed SST (ERSST) version 5 (2°×2°; Huang et al., 2017) and (2) the Hadley Centre Global SST (interpolated to 2°×2° before using; Rayner et al., 2003). Because the averaged SST data favors offsetting the inconsistent signal (or noise) between different datasets to certain extent (Hu et al., 2018). The low-level water vapor flux (WVF) is vertically integrated from surface to 600 hPa as follows:

$$WVF=\frac{1}{g}\int_{600}^{p_{s}} q\overrightarrow{V}dp$$

Here, *g* is the acceleration of Earth’s gravity, *p_s_* and *p* represent the surface pressure and air pressure, respectively, *q* is the specific humidity, and $\overrightarrow{V}$ indicates horizontal wind vector.

The season focused in this study is the boreal autumn (i.e., the seasonal mean of September-to-November, SON). The reason including that: (1) the extremely dry conditions in 2015 have led to severe fire activities over the MC during boreal autumn (Shawki et al. 2017); (2) a super ENSO was occurred in 2015, and El Nino is usually developing toward its peak in autumn; (3) the IOD events often reach its heyday during boreal autumn (e.g., Saji et al. 1999; Webster et al. 1999); (4) the ENSO and IOD properties have exhibited considerable changes in the context of greenhouse warming (e.g., Yang et al. 2018; Zhang et al. 2018); and (5) there are some studies on the relationship between ENSO/IOD and MC precipitation anomalies during boreal summer and winter (e.g., Feng & Chen, 2014; Jia et al., 2016), but it is relatively less during boreal autumn. Projections of the effects of ENSO-IOD change on the MC autumn precipitations are therefore inherently uncertain, thus which is a subject of profound-scientific interest in present study.

Unless noted otherwise, the monthly anomalies for each variable are calculated relatively to their respective monthly climatologic mean during 1979**–**2016. And then the seasonal anomalies of each variable are summed from the monthly anomalies correspondingly. Besides, for detection of interannual variation, all variables have been detrended.

As suggested by Aldrian & Susanto (2003), “the EOF is sensitive to the domain size, especially in a small area”. Hence care must be taken when choosing the decomposition method used to identify the leading modes of MC land precipitation. In this study, we use the REOF to capture the leading two modes of the interannual variability of monthly normalized and detrended precipitation anomalies over the MC for the following three reasons:

- Firstly, through a thorough statistical evaluation on the ability of EOF and REOF in reproducing a large number of stationary modes, Lian & Chen (2008) have pointed out that REOF analysis is overwhelmingly better than EOF in terms of accuracy and effectiveness, especially in capturing the localized patterns, such as the two types of El Niño.
- Secondly, considering that the local precipitation variances/variations often exhibit significant differentiations in different regions and different months (e.g., Zhang et al. 2016; Hu et al. 2018), applying a monthly normalization of the precipitation anomalies for each grid within the MC prior to the REOF or EOF analysis is more suitable and thus to be used in this study.
- Thirdly, we have also tested the relationship between the regional precipitation indices (i.e., the WMCPI and EMCPI shown in **Figure S1**) and the principle components of EOF and REOF. As shown in **Table S2**, the WMCPI is highly correlated to the RPC1 but not significantly correlated to the RPC2; meanwhile, the EMCPI is highly correlated to the RPC2 but not significantly correlated to the RPC1. This is consistent with the fact that the precipitation variations over the WMC and EMC are relatively independent with each other to a large extent since the covariance of WMCPI and EMCPI is less than 15%. However, both WMCPI and EMCPI are not only significantly correlated to PC1 but also significantly correlated to PC2 (**Table S2**). Besides, the PC1 contains not only the hybrid signals of EP ENSO and CP ENSO, but also the mixed signals of IOD and SIOD (see **Table S3** for details). Therefore, EOF decomposition has a tendency to produce hybrid modes especially for the PC1 (EOF1), which highly correlated with EMCPI (r=0.66) and WMCPI (r=0.86) at the same time. Accordingly, we use the REOF analysis in this study.

**Supplementary Note 1**

**About the normalized explained variance**

In Figures 1a and 1b, it is shown that the percent variances explained by REOF1 and REOF2 are 34.3% and 27.9%, respectively. But it should be noted that here the REOF analysis was based on a normalized precipitation anomalies (see **Supplementary Data and Methods**). In other words, here the REOF is applied to a correlation coefficient matrix rather than a covariance matrix. So the obtained eigenvectors are non-dimensional. The REOF (or EOF) analysis on the correlation coefficient matrix yields eigenvectors that correctly describe spatial distribution of the normalized anomalies, but not the amplitude of the anomalies (Wang et al., 2008).

Accordingly, here the obtained percent variance from the REOF is not the “realistic” explained variance. To obtain accurate explained variance and amplitude information, we calculated the locally percent variances explained by the RPC1 and RPC2 for reference (see the following **Figures S1e-S1f**). It is clear to see from **Figures S1e-S1f** that the percent variances explained by RPC1 and RPC2 up to a range from 35% up to about 60% (shading by red colors), with maximum centers over the western MC for REOF1, and over the eastern MC for REOF2.

**Supplementary Note 2**

**Testing the construction of EP-IOD and CP-SIOD indices**

Considering that the impacts from EP/CP ENSO index may be different from IOD/SIOD index for the two leading rotated principle components (i.e., RPC1 and RPC2), here we further use the multiple-regression to obtain their relative proportional relationships. Namely, given the normalized RPC1 = m*EPI + n*IOD, and based on the given conditions of (x + y = 1) and (x : y = m : n), we can get the values of x and y. Then the new combined index named as EP-IOD^b^, is put forward as below:

EP-IOD^b^ = x*EPI + y*IOD = 0.236661*EPI + 0.763339*IOD

Likewise, CP-SIOD^b^ = 0.513783*CPI + 0.486217*SIOD

In other words, the impact from EP ENSO is smaller than that from the IOD index for RPC1; whereas the weights from CP ENSO and SIOD are almost equal for RPC2. The corresponding results are shown in **Figure S9**. It is clear that the **Figure S9** well mirror the patterns shown in **Figure 1**, albeit with some weakening in the H200 anomalies over the tropical Indian Ocean and tropical Northeastern Pacific (**Figure S9e**) due to the smaller weight of EPI than IOD for EP-IOD^b^. In addition, there is no significant improvement for the correlation between RPC1 and EP-IOD^b^ (r = 0.73, **Figure S9a**), in contrast to that between RPC1 and EP-IOD (r = 0.71, **Figure 1a**); and there is no change in the correlation between CP-SIOD and RPC2 (**Figure S9b vs. Figure 1b**). For simplicity, we only use the EP-IOD and CP-SIOD indices in the main text.

**Supplementary References**

Aldrian E and Susanto RD. Identification of three dominant rainfall regions within Indonesia and their relationship to sea surface temperarure. *International Journal of Climatology* 2003; **23**, 1435–1452.

Adler RF, Huffman GJ, Chang A and Ferraro R, et al. The Version-2 Global Precipitation Climatology Project (GPCP) monthly precipitation analysis (1979–present). *Journal of Hydrometeorology*, 2003; **4**, 1147–1167.

Chen M, Xie P, Janowiak JE and Arkin PA. Global land precipitation: A 50-yr monthly analysis based on gauge observations. *Journal of Hydrometeorology*, 2002; **3**, 249–266.

Dee DP, Uppala SM, Simmons AJ and Berrisford P, et al. The ERA-Interim reanalysis: Configuration and performance of the data assimilation system. *Quarterly Journal of the Royal Meteorological Society*, 2011; **137**(656), 553–597. <https://doi.org/10.1002/qj.828>

Feng J and Chen W. Influence of the IOD on the relationship between El Niño Modoki and the East Asian-western North Pacific summer monsoon. *International Journal of Climatology* 2014; **34**, 1729–1736.

Harris I, Jones PD, Osborn TJ, Lister DH. Updated high-resolution grids of monthly climatic observations—The CRU TS3.10 dataset. *International Journal of Climatology*, 2014; 34, 623–642, <https://doi.org/10.1002/joc.3711>

Hu C, Chen D, Huang G and Yang S. Dipole types of autumn precipitation variability over the subtropical East Asia-western Pacific modulated by shifting ENSO. *Geophysical Research Letters*, 2018; **45**, 9123–9130. <https://doi.org/10.1029/2018GL078982>

Huang B, Thorne PW, Banzon VF, and Boyer T, et al. Extended Reconstructed Sea Surface Temperature version 5 (ERSSTv5), upgrades, validations, and intercomparisons. *Journal of Climate*, 2017; 30, 8179‒8205. <https://doi.org/10.1175/JCLI-D-16-0836.1>

Jia X, Ge J and Wang S. Diverse impacts of ENSO on wintertime rainfall over the Maritime Continent. *International Journal of Climatology,* 2016; 36: 3384–3397.

Lian T and Chen D. An evaluation of rotated EOF analysis and its application to tropical pacific SST variability. *Journal of Climate,* 2012; **25,** 5361–5373.

Lindzen RS and Nigam S. On the role of sea surface temperature gradients in forcing low-level winds and convergence in the tropics. *Journal of the Atmospheric Sciences*, 1987; **44** (17): 2418–2436.

Matsuura K and Willmott CJ. Terrestrial Precipitation: 1900-2017 Gridded Monthly Time Series (Version 5.01), 2018; <http://climate.geog.udel.edu/~climate/html_pages/Global2017/README.GlobalTsP2017.html>

Rayner NA, Parker DE, Horton EB, and Folland CK, et al. Global analyses of sea surface temperature, sea ice, and night marine air temperature since the late nineteenth century. *Journal of Geophysical Research*, 2003; **108**, 4407. <https://doi.org/10.1029/2002JD002670>

Shawki D, Field RD and Tippett MK *et al*. Long-lead prediction of the 2015 fire and haze episode in Indonesia. *Geophysic Research Letters* 2017; **44**:9996–10005.

Saji NH, Goswami BN, Vinayachandran PN and Yamagata T. A Dipole mode in the tropical Indian Ocean. *Nature* 1999; **401**: 360–363.

Wang B, Wu Z, Li J, Liu J, Chang, Ding Y, and Wu G. How to Measure the Strength of the East Asian Summer Monsoon. *Journal of Climate*, 2008; **21**, 4449–4463, <https://doi.org/10.1175/2008JCLI2183.1>

Webster PJ, Moore AM, Loschnigg JP and Leben RR. Coupled ocean-atmosphere dunamics in the Indian Ocean during 1997-98. *Nature* 1999; **401**: 356–360.

Yang S, Li Z and Yu J-Y *et al*. El Niño-Southern Oscillation and its impact in the changing climate. *National Science Review* 2018; **5**:840–857.

Zhang L, Du Y and Cai W. A spurious positive Indian Ocean Dipole in 2017, *Science Bulletin* 2018; **63**:1170–1172.

Zhang T, Yang S and Jiang X *et al*. Roles of remote and local forcings in the variation and prediction of regional Maritime Continent rainfall in wet and dry seasons. *Journal of Climate* 2016; **29**:8871–8879.

Zhang Z and Zhou T. Drought over East Asia: A Review. *Journal of Climate*, 2015; **28**, 3375–3398.

Zhang Z, Sun X and Yang X. Understanding the Interdecadal Variability of East Asian Summer Monsoon Precipitation: Joint Influence of Three Oceanic Signals. *Journal of Climate*, 2018; **31**, 5485–5506.

**Supplementary Tables & Figures**

**Table S1** Description of the data sources used in this study.

| Date sets (resolution) | Download Websites | References |
| --- | --- | --- |
| NOAA PREC/L (1°×1°)  NOAA PREC/L (0.5°×0.5°)  GPCP V2.3 (2.5°×2.5°)  Delaware Precipitation (0.5°×0.5°)  CRU Precipitation (0.5°×0.5°)  NOAA ERSST V5 (2°×2°)  Hadley Global SST (1°×1°)  ERA-Interim (2.5°×2.5°) | <ftp://ftp.cdc.noaa.gov/Datasets/precl/1.0deg/precip.mon.mean.1x1.nc>  <ftp://ftp.cdc.noaa.gov/Datasets/precl/0.5deg/precip.mon.mean.0.5x0.5.nc>  <https://www.esrl.noaa.gov/psd/data/gridded/data.gpcp.html>  <ftp://ftp.cdc.noaa.gov/Datasets/udel.airt.precip/precip.mon.total.v501.nc>  <http://data.ceda.ac.uk/badc/cru/data/cru_ts/cru_ts_4.03/data/pre>  <https://www.esrl.noaa.gov/psd/data/gridded/data.noaa.ersst.v5.html>  <https://www.metoffice.gov.uk/hadobs/hadisst/data/download.html>  <https://www.esrl.noaa.gov/psd/data/index.html> | Chen et al. (2002)  Chen et al. (2002)  Adler et al. (2003)  Matsuura & Willmott (2018)  Harris et al. (2014)  Huang et al. (2017)  Rayner et al. (2003)  Dee et al. (2011) |

**Table S2** Correlation matrix between two regional MC precipitation indices and the principle components of the leading two REOF and EOF results. |Correlations| with p<0.05 are in bold.

| Correlation | RPC1 | RPC2 | PC1 | PC2 |
| --- | --- | --- | --- | --- |
| EMCPI  WMCPI | -0.06  **0.90** | **0.94**  0.27 | **0.66**  **0.86** | **0.67**  **0.38** |

**Table S3** Correlation matrix between the principle components of the leading two EOF results and different ENSO and IOD indices. |Correlations| with p<0.05 are in bold.

| Correlation | EPI | CPI | IOD | SIOD | EPI+CPI | Nino3.4b |
| --- | --- | --- | --- | --- | --- | --- |
| PC1  PC2 | **0.72**  0.07 | **0.64**  0.26 | **0.76**  0.21 | **0.42**  **0.44** | **0.84**  0.10 | **0.81**  0.07 |


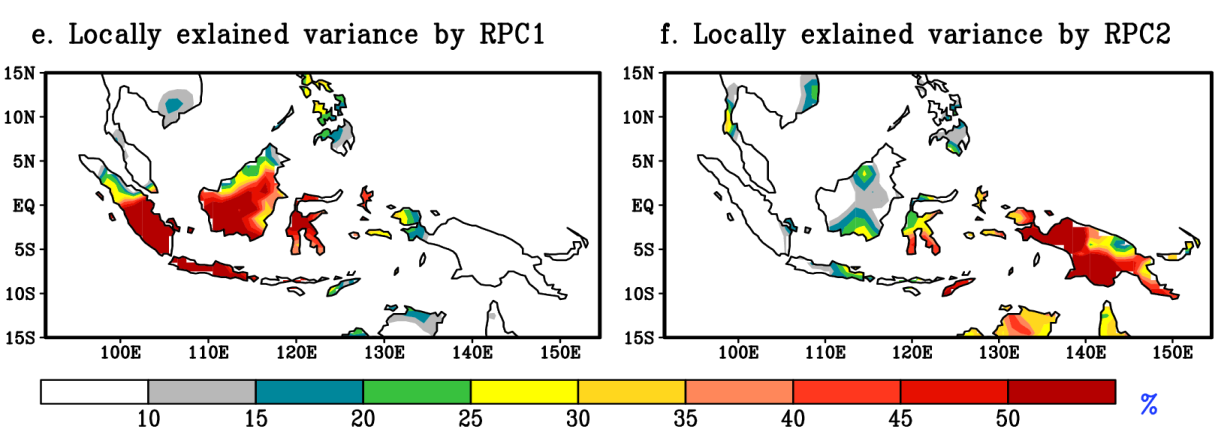

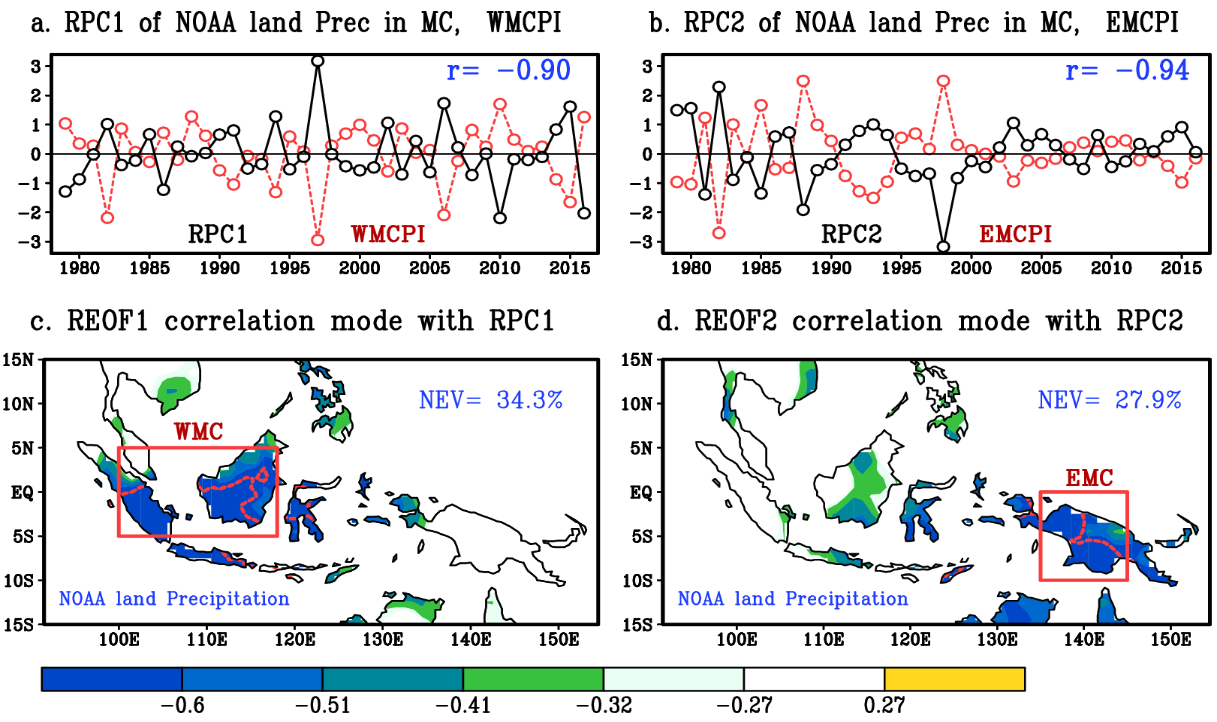


**Figure S1.** REOF results of the normalized and detrended NOAA land precipitation anomalies (1°×1°) over the MC region (95°E–145°E, 11°S–9°N) during SON of 1979–2016. (a) Normalized and detrended RPC1 (black line). (c) REOF1 correlation mode of precipitation anomalies associated with RPC1 shown in (a); here the red dotted lines indicate the |correlation| up to 0.70. (b) and (d) as in (a) and (c) except for RPC2 and REOF2. Also shown in (a) and (b) are the WMC and EMC precipitation indices (i.e., WMCPI, EMCPI) that are defined as the seasonal area-mean of monthly normalized and detrended NOAA land precipitation anomalies over the red box-region outlined in (c) 100–118°E, 5°S–5°N and (d) 135–145°E, 10°S–0. The correlation between RPC1 and WMCPI (RPC2 and EMCPI) is high up to -0.90 (-0.94), far exceeding the 99.9% confidence level and suggesting that the two REOF modes are physically significant. Shown in (e) and (f) are the locally percent variances explained by RPC1 and RPC2.


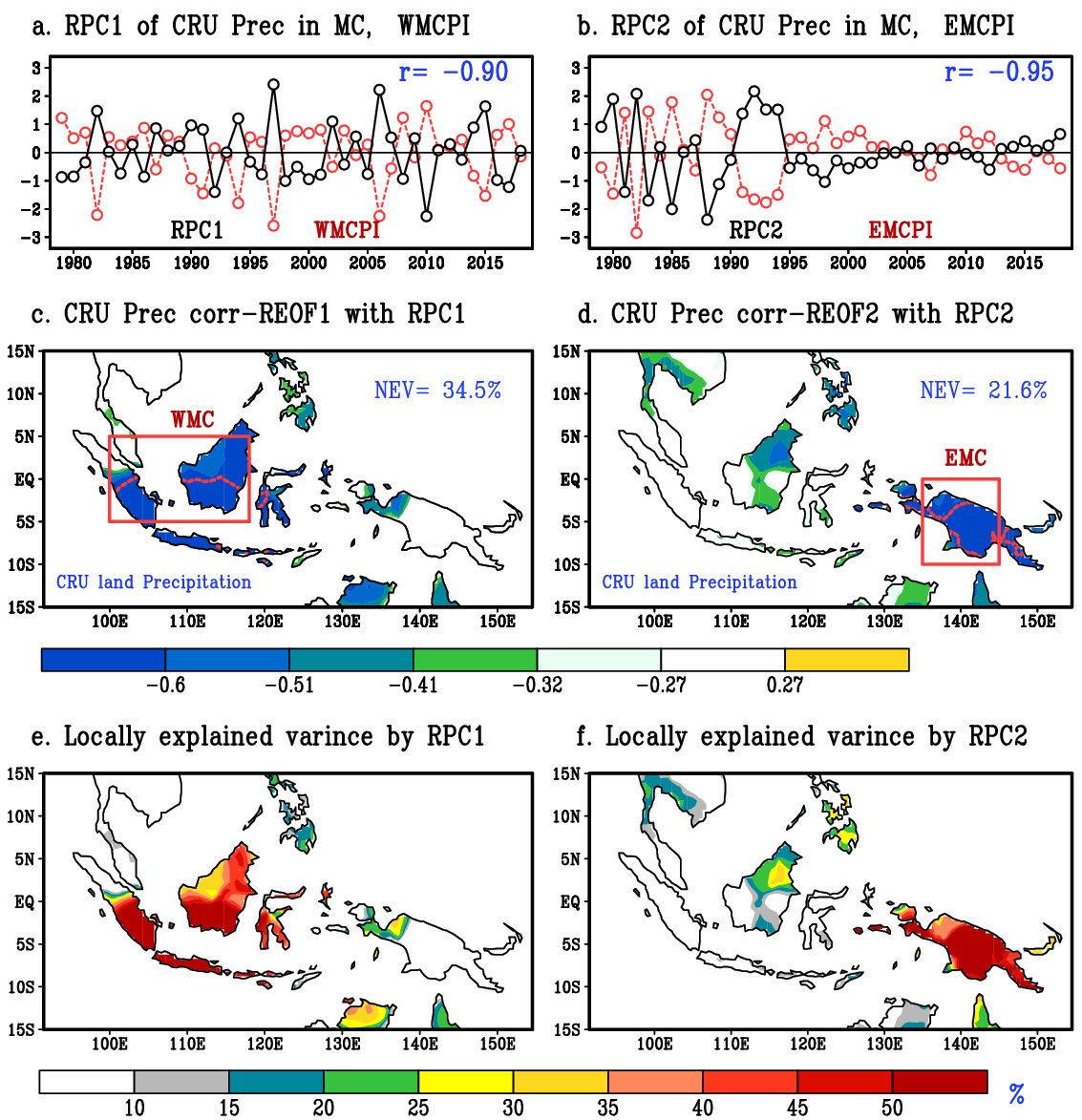


**Figure S2.** Same as Figure S1, except for the land precipitation datasets from the Climatic Research Unit (CRU, 0.5°×0.5°) at the University of East Anglia during 1979–2018.


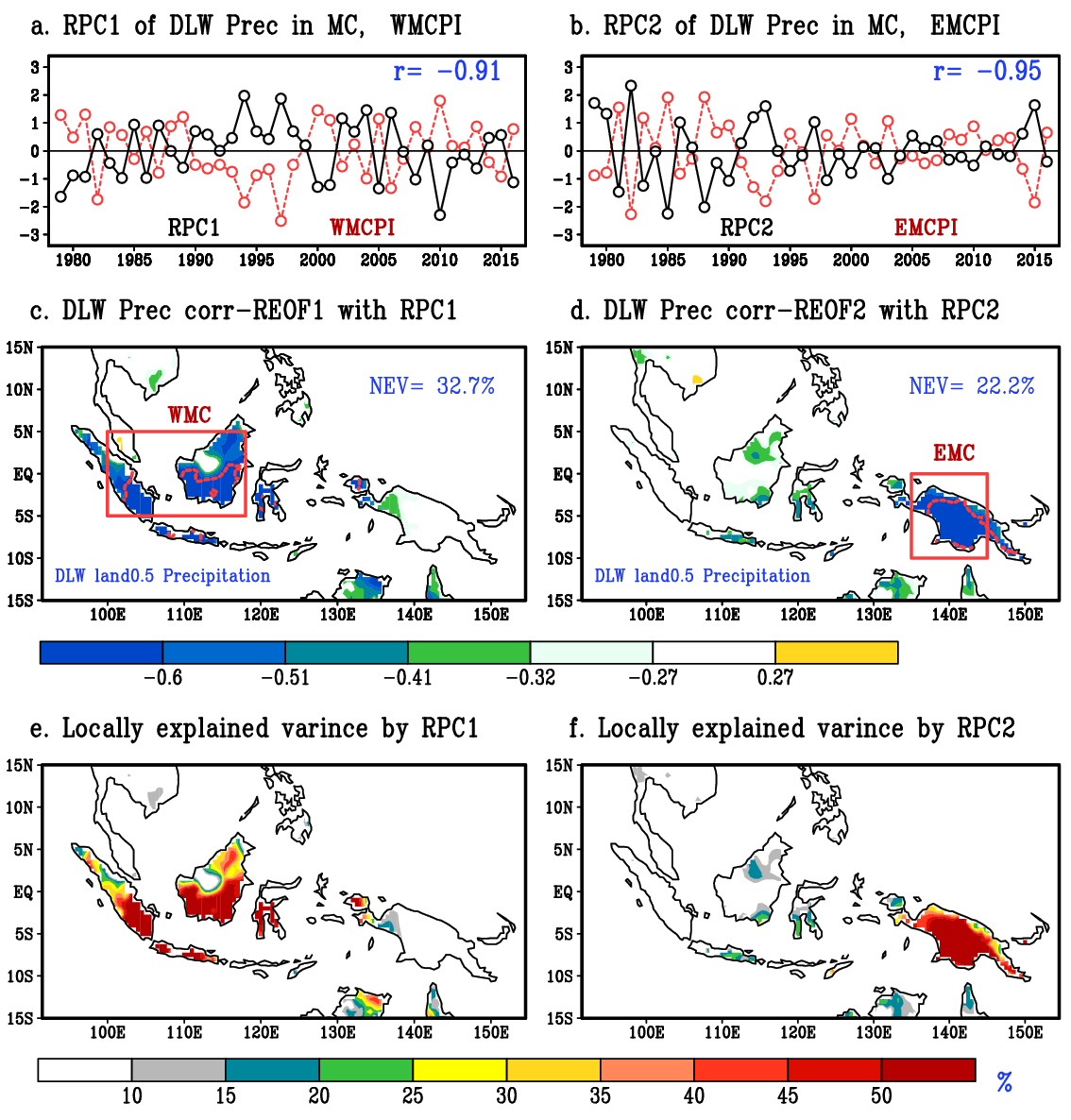


**Figure S3.** Same as Figure S1, except for the land precipitation datasets from the University of Delaware (DLW, 0.5°×0.5°) during 1979–2016.


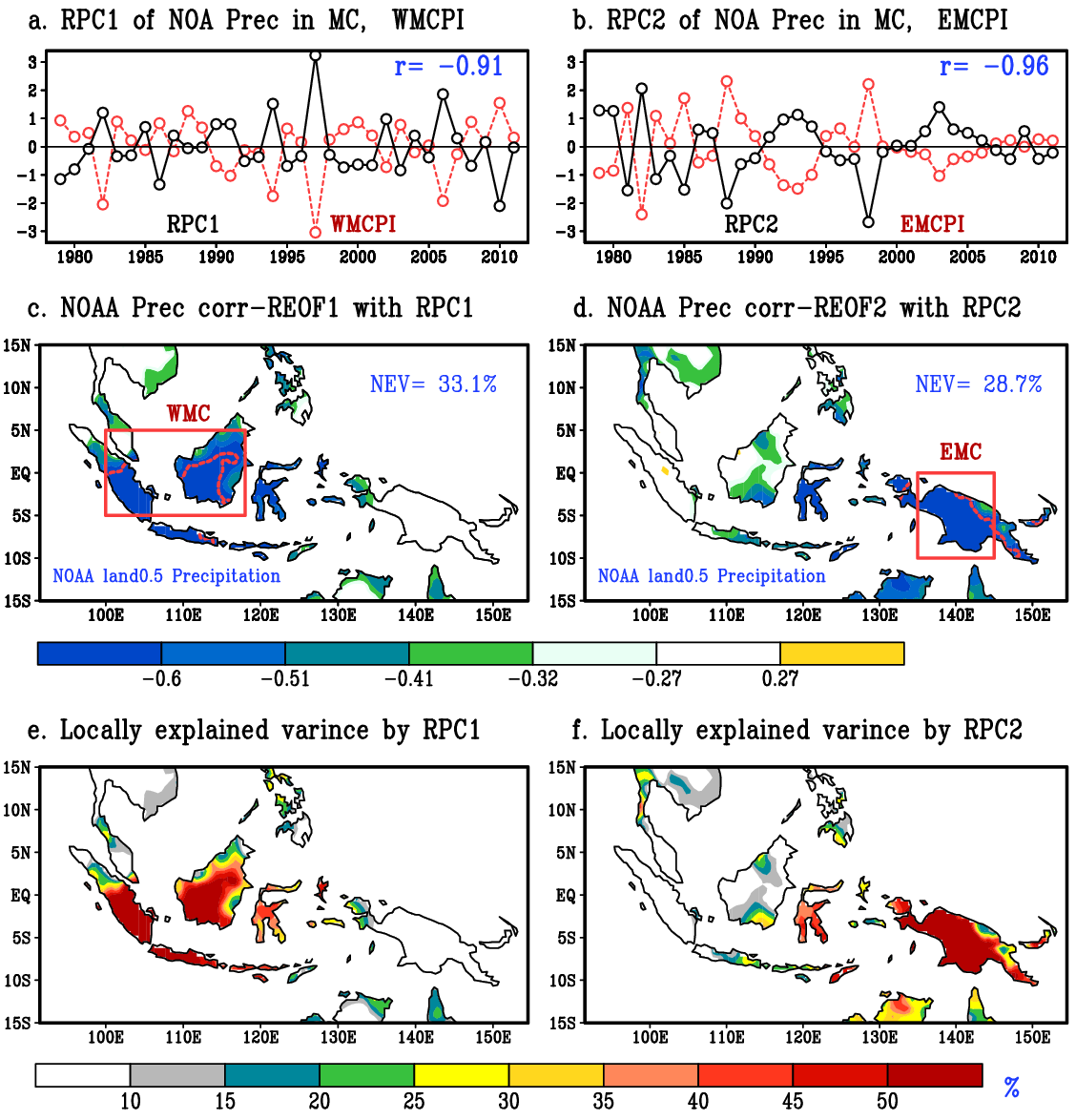


**Figure S4.** Same as Figure S1, except for the NOAA land precipitation datasets (version of 0.5°×0.5°) during 1979–2011. Since the version of 0.5°×0.5° is only updated to 2012/01 now, thus not be preferentially shown in the main text.


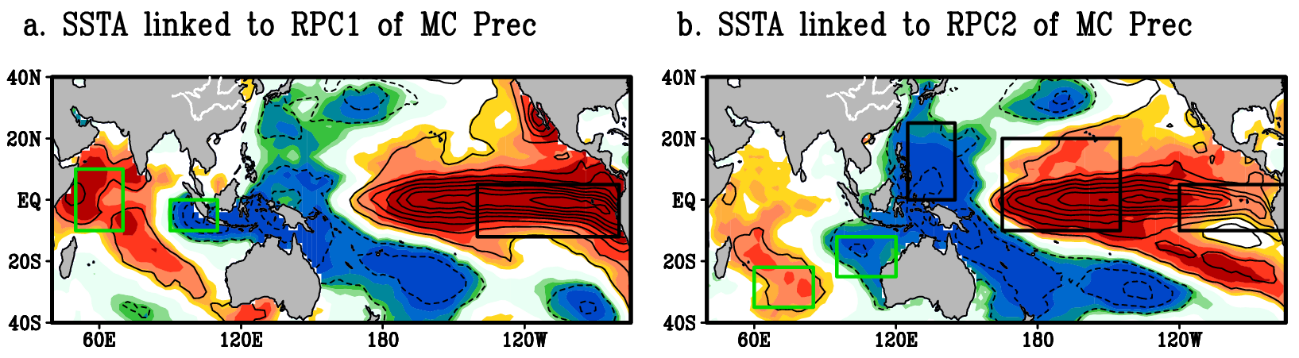

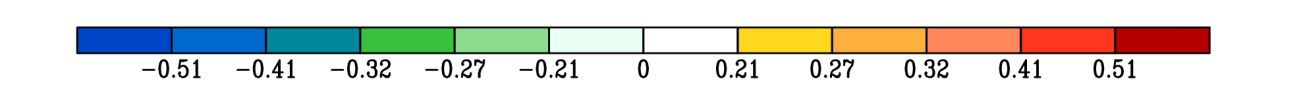


**Figure S5.** Indo-Pacific SST patterns (shadings are used for correlation, and contours are regression with an interval 0.1 K) associated with (a) RPC1 and (b) RPC2 of NOAA land precipitation anomalies (1°×1°) over the MC.


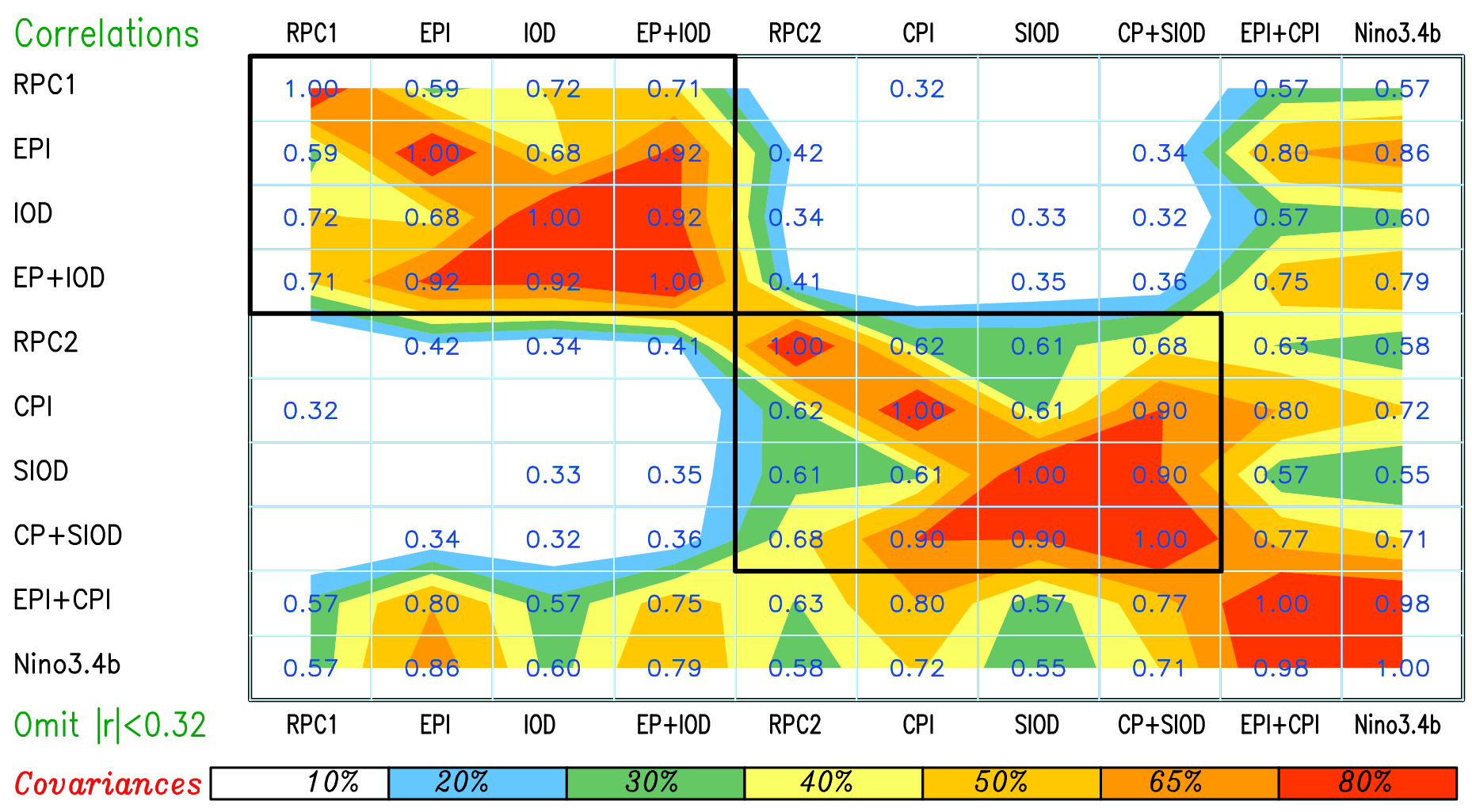


**Figure S6.** Correlation matrix of RPC1, RPC2, and two types of ENSO and IOD indices as well as EP-IOD, CP-SIOD, EPI+CPI and Nino3.4 (all indices are normalized and detrended). Here only show the correlation exceeding the estimated 95% confidence level. Shading areas indicate the explained covariances.


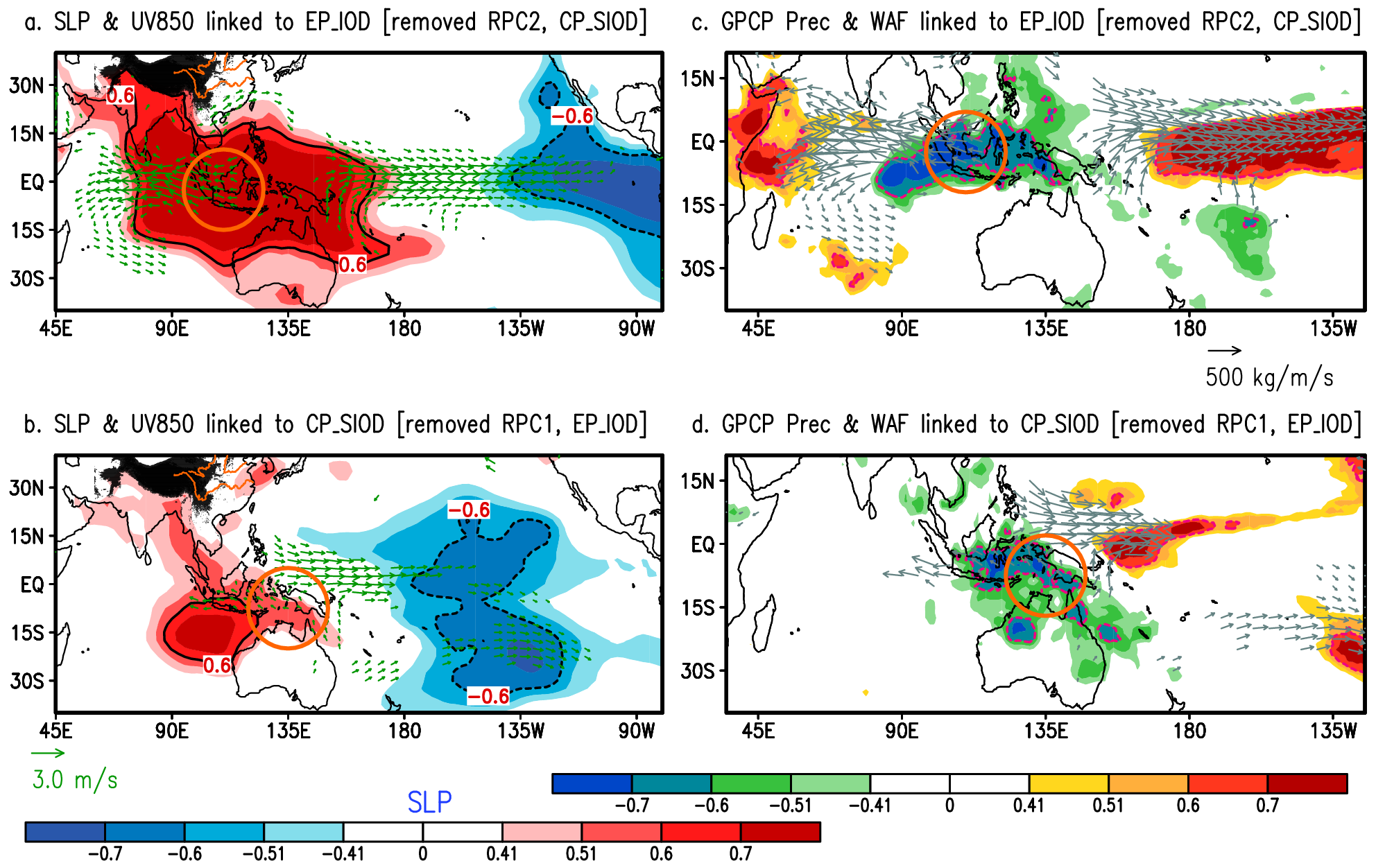


**Figure S7.** Patterns of (a) SLP (shading) & low-level wind (UV850, vector) and (c) GPCP precipitation (shading) & low-level water vapor flux (WVF, vector, vertical integrated from surface to 600hPa) linked to the EP-IOD after removing the RPC2 and CP-SIOD. (b) and (d) are same as in (a) and (c) except for the CP-SIOD after removing the RPC1 and EP-IOD. Here the shadings and vectors are correlations and regressions, respectively. Thick-lines indicate the |correlations| above 0.60. Only vectors exceeding the estimated 99% confidence level are shown. The orange circles highlight the relative MC position as a reference system. Such patterns highlight the importance of surface pressure (i.e., SLP) gradients due to Indo-Pacific SST gradients to low-level wind and convergence or divergence via the so-called Lindzen-Nigam mechanism (Lindzen and Nigam, 1987).


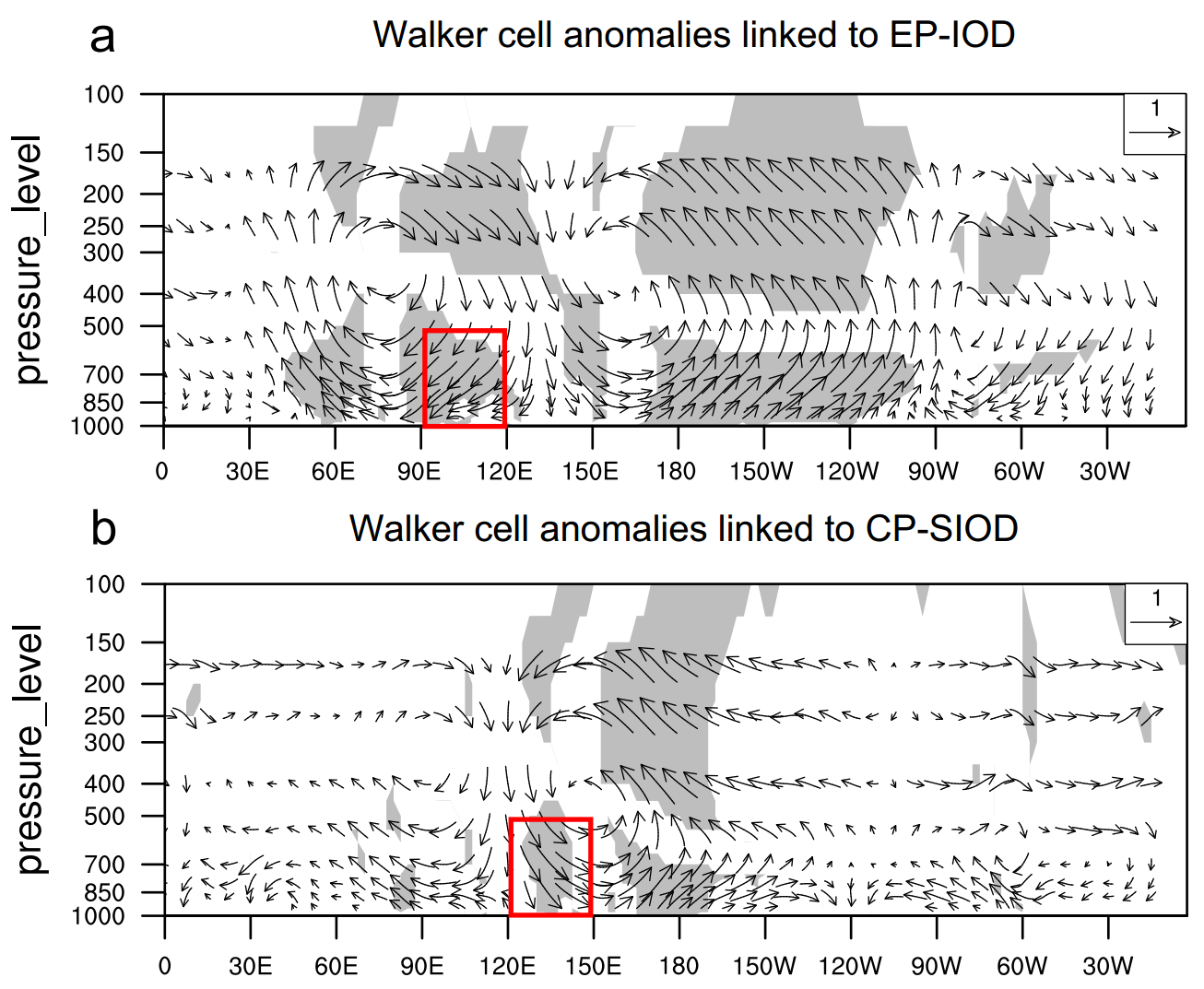


**Figure S8.** Same as in Figures 1c and 1d except for the Walker circulation patterns (averaged between 10°S-10°N) associated with (a) EP-IOD (after removing RPC2) and (b) CP-SIOD (after removing RPC1). Shading areas indicate the correlation exceeding the estimated 95% confidence level.


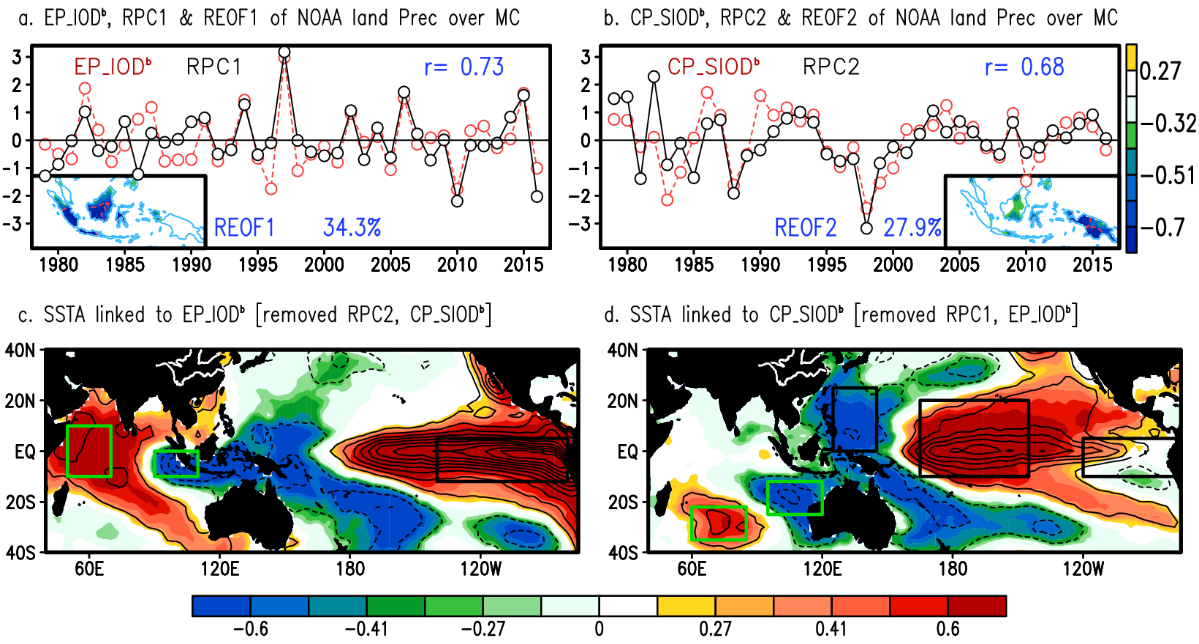

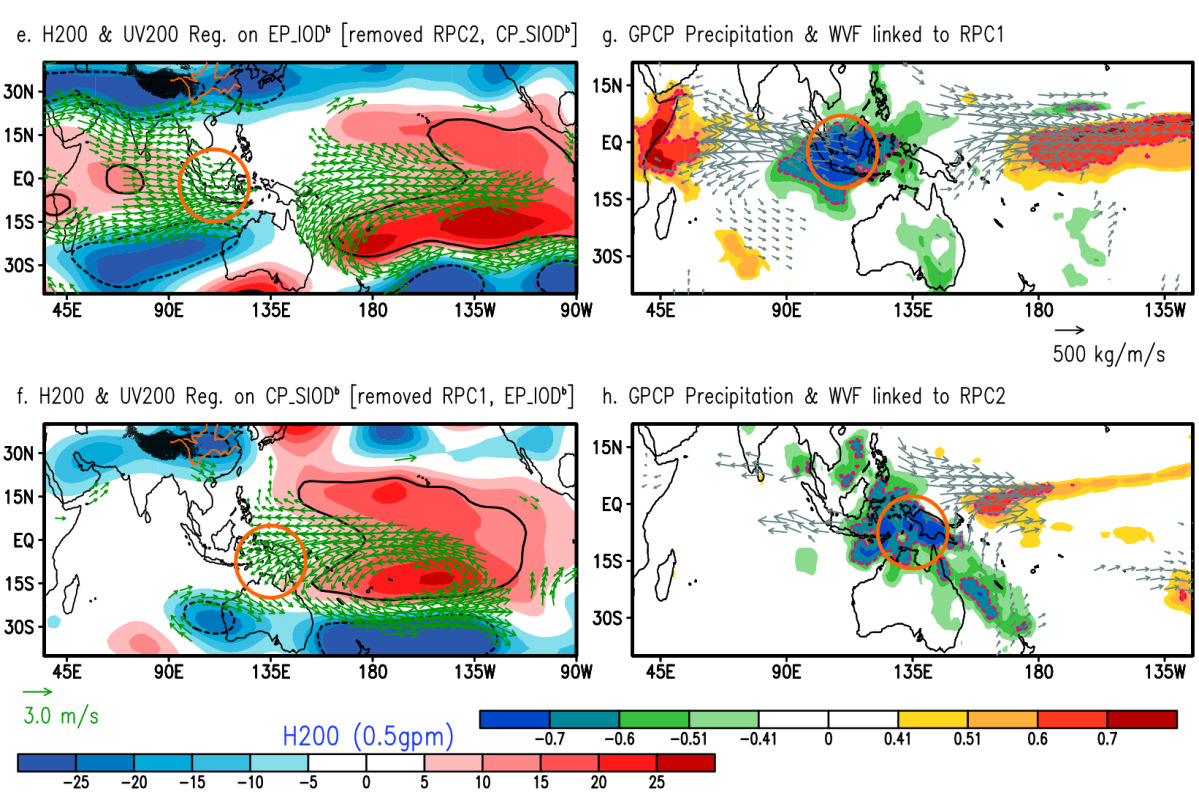


**Figure S9.** Same as Figure 1, except the EP-IOD^b^ = 0.236661*EPI + 0.763339*IOD, and the CP-SIOD^b^ = 0.513783*CPI + 0.486217*SIOD. Here the proportional relationships are obtained from multiple-regression for RPC1 and RPC2, respectively.
